# Dysregulation of X Chromosome Inactivation in High Grade Ovarian Serous Adenocarcinoma

**DOI:** 10.1371/journal.pone.0118927

**Published:** 2015-03-05

**Authors:** Jun Kang, Hee Jin Lee, Jiyoung Kim, Jae Jun Lee, Lee-so Maeng

**Affiliations:** 1 Department of Hospital Pathology, Inchun St. Mary’s hospital, College of Medicine, The Catholic University of Korea, Inchun, Republic of Korea; 2 Department of Pathology, University of Ulsan College of Medicine, Asan Medical Center, Seoul, Republic of Korea; Princess Margaret Cancer Centre, CANADA

## Abstract

**Background:**

One of the two copies of the X chromosome is randomly inactivated in females as a means of dosage compensation. Loss of X chromosome inactivation (XCI) is observed in breast and ovarian cancers, and is frequent in basal-like subtype and *BRCA1* mutation-associated breast cancers. We investigated the clinical implications of the loss of XCI in ovarian cancer and the association between the loss of XCI and *BRCA1* dysfunction.

**Materials and Methods:**

We used open source data generated by The Cancer Genome Atlas (TCGA) Genome Data Analysis Centers. Ward’s hierarchical clustering method was used to classify the methylation status of the X chromosome.

**Results:**

We grouped 584 high grade serous ovarian adenocarcinomas (HG-SOA) according to methylation status, loss of heterozygosity and deletion or gain of X chromosome into the following five groups: preserved inactivated X chromosome (Xi) group (n = 175), partial reactivation of Xi group (n = 100), p arm deletion of Xi group (n = 35), q arm deletion of Xi group (n = 44), and two copies of active X group (n = 230). We found four genes (*XAGE3*, *ZNF711*, *MAGEA4*, and *ZDHHC15*) that were up-regulated by loss of XCI. HG-SOA with loss of XCI showed aggressive behavior (overall survival of partial reactivation of Xi group: HR 1.7, 95% CI 1.1–2.5, two copies of active X group: HR 1.4, 95% CI 1.0–1.9). Mutation and hypermethylation of *BRCA1* were not frequent in HG-SOA with loss of XCI.

**Conclusions:**

Loss of XCI is common in HG-SOA and is associated with poor clinical outcome. The role of *BRCA1* in loss of XCI might be limited. XCI induced aberrant expression of cancer-testis antigens, which may have a role in tumor aggressiveness.

## Introduction

One of the two copies of the X chromosome is randomly inactivated in females as a means of dosage compensation. Random X chromosome inactivation (XCI) is initiated by an X-inactive specific transcript (*XIST*), a 17 kilobase noncoding RNA, and processed by histone modification and promoter methylation, resulting in heterochromatin formation [1]. Heterochromatin is tightly packed DNA and prevents gene expression.

Loss of XCI is thought to have an oncogenic effect in hematologic malignancy in mice [2]. It is also observed in human breast and ovarian cancers [3–5]. Cancer cells with loss of XCI have two active copies of the X chromosome. It was suggested that the presence of the two active copies increases the expression of the oncogenes located on the X chromosome. However, it is unknown which genes are affected by loss of XCI.

Chromatin segregation errors that move both active X chromosomes (Xa) to the same daughter cell during mitosis are thought to be a main mechanism of loss of XCI in cancer cells. Breast cncer 1, early onset (BRCA1) inactivation is another mechanism of the loss of XCI. However, there is a debate whether BRCA1 dysfunction directly induces the loss of XCI or if does so indirectly due to increasing the probability of a chromosome segregation error [1, 6–10].

Loss of XCI is frequent in basal-like subtype and *BRCA1* mutation-associated breast cancers, which show aggressive behavior [11–13]. However, the clinical implications of the loss of XCI in ovarian cancer are largely unknown. We investigated the clinical implications of the loss of XCI in ovarian cancer and the association between the loss of XCI and *BRCA1* dysfunction.

## Materials and Methods

### Data acquisition

We used open source data generated by The Cancer Genome Atlas (TCGA) Genome Data Analysis Centers. High grade serous ovarian adenocarcinoma (HG-SOA) data on DNA methylation, *XIST* expression levels, copy number variation (CNV), loss of heterozygosity (LOH), mRNA expression, and clinical data, including age, race, and ethnicity, were downloaded from the Broad genome data analysis center (GDAC) Firehose website (http://gdac.broadinstitute.org/). Names of files downloaded from firehose website are described in S1 Text. Data on *BRCA1* mutations were obtained from the c-bio portal for cancer genomics website (http://www.cbioportal.org/public-portal/). Data on survival, tumor stage, and grade were gathered using the CGDS-R package which is a package of R for querying the Cancer Genomics Data Server and is hosted by the Computational Biology Center at Memorial-Sloan-Kettering Cancer Center [14]. Data on *XIST* expression levels in normal endometrium and clinical data were downloaded from the TCGA Data Portal (https://tcga-data.nci.nih.gov/tcga/tcgaHome2.jsp).

### Brief description of the data

The beta values for DNA methylation status were estimated using the Illumina Infinium HumanMethylation27 array. The beta value is an estimate of the ratio of intensities between methylated and unmethylated alleles. Segmented copy number was estimated by log2 of the ratio of total intensity of the tumor and the normal tissue using Agilent 1M array. Segmented LOH was estimated by difference in allelic ratio (delta B) between tumor and normal tissue at loci where the normal sample is genotyped as heterozygous using 1MDuo SNP arrays. Large delta B values mean LOH. Normalized RNA-Seq by Expectation Maximization (RSEM) was used for estimating *XIST* expression [15]. Data on RSEM were generated using IlluminGA_RNASeqV2 or IlluminaHiSeq_RNASeqV2 platforms [16, 17]. DNA sequencing of the exome was done with Illumina GAIIx or ABI SOLiD platforms. AgilentG4502A was used to estimate z-score of mRNA expression. Detailed information about participants, specimen processing, and analysis of each molecular profiling platform have been described elsewhere [18].

### Ethics statement

All data used in this study were obtained from TCGA. The National Cancer Institute and National Human Genome Research Institute work with physicians who collect tissue for TCGA to gain approval with local institutional review boards (http://cancergenome.nih.gov/abouttcga/policies/informedconsent).

### Clustering analysis of X chromosome methylation

We selected 147 methylation array probes that showed large variations in beta values (larger than the third quartile) among probes targeting CpG islands in the X chromosome. CpG islands are major sites of methylation during XCI [19]. Ward’s hierarchical clustering method was used to classify the methylation status of X chromosomes.

### Segmental deletion or gain and LOH of X chromosome

The status of segmental deletion or gain and LOH of X chromosome were visualized using Integrative Genomics Viewer (IGV) [20, 21].

### Genes regulated by loss of X chromosome inactivation

Up-regulated genes by loss of XCI were investigated. To find hypomethylated genes in the HG-SOA with loss of XCI, we selected the genes which the difference of the median beta values were larger than 0.2. mRNA expression of the selected differently-methylated genes was compared using the Wilcox rank sum test. We selected genes for which the difference of the mRNA expression levels were larger than 0.4 and the p-value was less than 0.001. Gene set enrichment analysis (GSEA) was done to identify the cytogenetic locations where were regulated by the loss of XCI [22, 23]. The positional gene sets were used under default setting.

### Global methylation

We selected 4879 methylation array probes that showed large variations in beta values (larger than the third quartile) among probes targeting somatic chromosomes. The median beta value of each sample was compared among X chromosome methylation clusters using ANOVA and Tukey’s test.

### *BRCA1* methylation classification

The DNA methylation status of *BRCA1* was classified as hypermethylation or hypomethylation by k-means clustering methods. This method has been described in detail elsewhere [24].

### Clinical data and survival

Patient and tumor characteristics were compared among the groups of X chromosomes via methylation clustering analysis. The significance was estimated using Fisher’s exact test for categorical variables and analysis of variance for continuous variables. Univariate and multivariate survival analyses were performed with Log-rank and Cox regression tests, respectively.

## Results

### *XIST* expression in normal female tissues

To determine the baseline level of *XIST* expression in normal tissue, we analyzed *XIST* expression data from normal tissues. *XIST* expression was relatively constant across different female normal tissues (S1 Fig.). Because normal ovarian tissue was not available in TCGA data, we used *XIST* expression data of normal endometrial tissue (median log-transformed normalized RSEM: 9.3, range: 8.8–10.0). We considered *XIST* expression to be low when its levels in HG-SOA were lower than the lowest value observed among normal endometrial tissue.

### Clustering of X chromosome methylation

**Fig 1:**
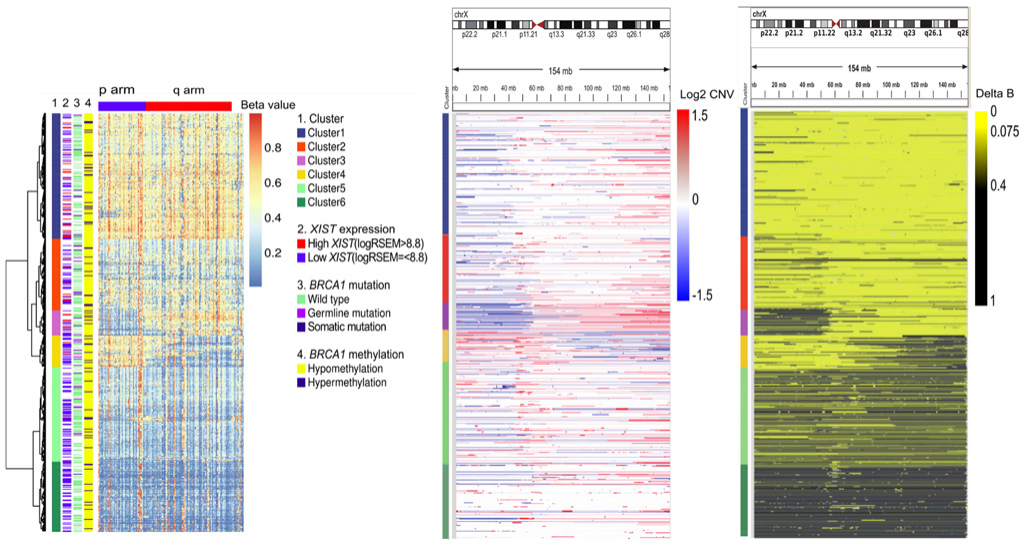
Dysregulation of X chromosome inactivation in high grade serous ovarian adenocarcinoma. A) 584 high grade serous ovarian adenocarcinomas were clustered by X chromosome methylation. Each column represents selected methylation probes sorted by location on the X chromosome. B) Red and blue represent segmental gain and deletion, respectively. C) Black and yellow represent segmental loss of heterozygosity and retained heterozygosity, respectively. A-C) Each row represents clustered samples.

A total of 584 HG-SOAs were classified into six clusters according to X chromosome methylation (Fig. 1A). Cluster 1 showed heterozygous X chromosomes (Fig. 1C) with hypermethylation on most of the included loci on the X chromosome (Fig. 1A), as well as high *XIST* expression (Table 1). These results meant that the HG-SOAs of cluster 1 had both Xa and Xi. Clusters 3 and 4 showed selective p arm and q arm hypomethylation, respectively (Fig. 1A). This selective hypomethylation corresponded to deletion and LOH of the X chromosome in the p and q arms (Fig. 1B and 1C). The hypomethylation in clusters 3 and 4 suggested that the deletion occurred in the inactive X chromosome (Xi), but not Xa. Clusters 3 and 4 also showed q and p arm gain, respectively (Fig. 1B). However, it is not clear if the gain occurred in Xi or Xa. Cluster 2 showed heterozygous X chromosomes (Fig. 1C) and high *XIST* expression (Table 1) like cluster 1. These results meant the HG-SOAs of cluster 2 had both Xa and Xi. But cluster 2 showed partial hypomethylation on both the p and q arms of the X chromosome unlike cluster 1 (Fig. 1A). The partial hypomethylation represented partial reactivation of Xi, thus resulting in an increased dosage of Xa. Clusters 5 and 6 showed LOH at X chromosomes (Fig. 1C) but not deletion of a whole X chromosome (Fig. 1B). These results meant that clusters 5 and 6 had copy-neutral LOH. Cluster 5 and 6 also showed hypomethylation at most loci on the X chromosome (Fig. 1A) and low *XIST* expression (Table 1). These results suggested that HG-SOAs of cluster 5 and 6 had uniparental two copies of an active X chromosome. Each cluster was designated as the preserved Xi group in the case of cluster 1 (n = 175), partial reactivation of the Xi (Xa^+^) group for cluster2 (n = 100), p arm deletion of the Xi (Xi-p^-^) group for cluster 3 (n = 35), q arm deletion of the Xi (Xi-q^-^) group for cluster 4 (n = 44), and two copies of the Xa (two Xa) group for clusters 5 and 6 (n = 230). The result of the post-hoc analysis of *XIST* expression is summarized in S1 Table.

**Table 1:** *XIST* expression and *BRCA1* mutations and methylation.

	Cluster 1 (Preserved Xi)	Cluster 2 (Partial reactivation of Xi)	Cluster 3 (p arm deletion of Xi)	Cluster 4 (q arm deletion of Xi)	Cluster 5 (Two copies of Xa)	Cluster 6 (Two copies of Xa)	*P* value
*XIST* expression, log transformed RSEM (SD) (*N* = 262)*	8.59 (0.87)	8.18 (0.70)	7.99 (1.23)	7.67 (1.10)	6.77 (0.89)	5.92 (0.96)	<0.001^†^
*BRCA1* mutation (*N* = 316)*							<0.001^†^ ^‡^
Wild type	79 (89.8%)	47 (83.9%)	16 (84.2%)	16 (66.7%)	71 (89.9%)	49 (98.0%)	
Germline mutation	4 (4.5%)	6 (10.7%)	2 (10.5%)	8 (33.3%)	7 (8.8%)	0 (0%)	
Somatic mutation	5 (5.7%)	3 (5.4%)	1 (5.3%)	0 (0%)	1 (1.3%)	1 (2.0%)	
*BRCA1* methylation (*N* = 584)*							0.712
Hypomethylation	152 (86.9%)	89 (89.0%)	29 (82.9%)	37 (84.1%)	119 (90.2%)	88 (89.8%)	
Hypermethylation	23 (13.1%)	11 (11.0%)	6 (17.1%)	7 (15.9%)	13 (9.8%)	10 (10.2%)	

*, sample size is different in each analysis

†, statistically significant

‡, *P*-value of fisher’s exact test to compare frequency of *BRCA1* germline mutations in cluster 4 (33.3%) with other clusters (5.9%)

### Genes regulated by loss of X chromosome inactivation

We found four genes including *X antigen family*, *member 3* (*XAGE3*), *zinc finger protein 711* (*ZNF711*), *melanoma antigen family A*, *4* (*MAGEA4*), and *zinc finger*, *DHHC-type containing 15* (*ZDHHC15*), which showed significantly higher expression in the two Xa group than in the preserved Xi group (S2 Fig.). GSEA did not identify significantly over-expressed cytogenetic locations of X chromosome in the two Xa group than the preserved Xi group (S2 Table). Contrary to the expectation, the preserved Xi group was more enriched higher expression of genes located at Xp22 and Chr9q22 than the two Xa group (S3 Table).

### Global methylation

**Fig 2:**
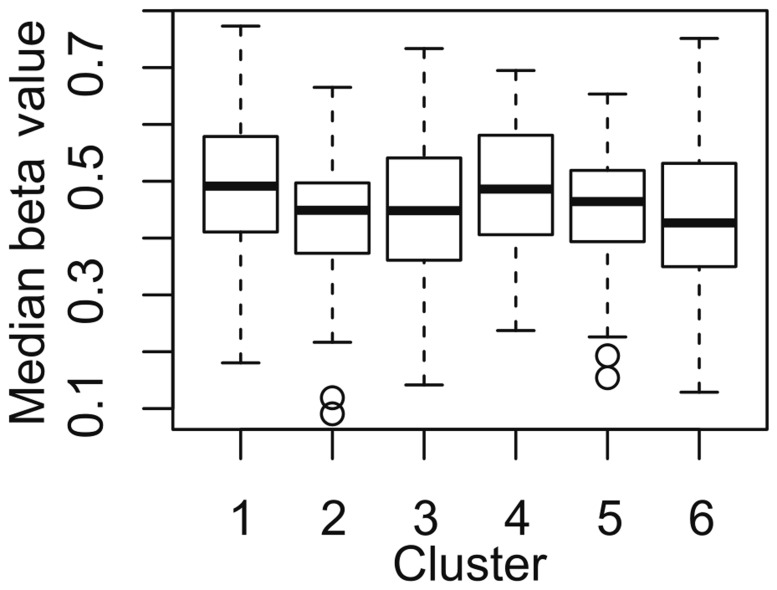
Median beta values of somatic chromosomes. Box plots represent the distribution of median beta values of somatic chromosomes in each cluster. The top and bottom of the box represent the 75^th^ and 25^th^ percentile, the line in the box represents median, the whiskers present minimum and maximum, and the points represent the outliers.

Clusters 2 and 6 showed lower median beta values of methylation on somatic chromosomes (overall *P*-value <0.001, Fig. 2). The results of the post-hoc test are summarized in S4 Table.

### *BRCA1* mutation and hypermethylation

*BRCA1* mutation information was available for 316 HG-SOAs. Mutation and promoter lesion hypermethylation of *BRCA1* were not frequent in HG-SOAs with a loss of XCI (Xa^+^ and two Xa groups). The frequency of germline mutations in *BRCA1* was significantly higher in the Xi-q^-^ group (Table 1).

### Clinical and tumor characteristics

Patients in the Xa^+^ and two Xa groups were significantly older than those in the other groups (Table 2). There were no significant differences in race, ethnicity, tumor stage, grade, or residual tumor size among the groups. The results of the post-hoc test for difference of patient age among the groups are summarized in S5 Table.

**Table 2:** Clinical and tumor characteristics.

	Preserved Xi	p arm deletion of Xi	q arm deletion of Xi	Partial reactivation of Xi	Two copies of Xa	*P* value
Age (*N* = 566)*	57.6 (11.6)	58.1 (12.0)	54.9 (8.5)	62.2 (11.9)	61.5 (11.4)	<0.001^†^
Race (*N* = 534)*						0.997
American Indian or Alaska native	1 (0.6%)	0 (0.00%)	0 (0.00%)	0 (0.00%)	2 (1.0%)	
Asian	5 (3.2%)	1 (3.0%)	2 (4.8%)	3 (3.3%)	7 (3.3%)	
Black or African American	7 (4.4%)	1 (3.0%)	1 (2.3%)	6 (6.5%)	9 (4.3%)	
Native Hawaiian or other Pacific islander	0 (0%)	0 (0%)	0 (0%)	0 (0%)	1 (0.5%)	
White	145 (91.8%)	31 (93.9%)	39 (92.9%)	83 (90.2%)	190 (90.9%)	
Ethnicity (*N* = 340)*						0.3
Hispanic or Latino	3 (2.8%)	2 (8.0%)	1 (4.5%)	3 (4.7%)	2 (1.6%)	
Not Hispanic or Latino	104 (97.2%)	23 (92.0%)	21 (95.5%)	61 (95.3%)	120 (98.4%)	
AJCC stage (*N* = 484)*						0.534
II	9 (6.5%)	0 (0.00%)	2 (5.1%)	5 (6.1%)	8 (4.1%)	
III	109 (79.6%)	24 (80.0%)	31 (79.5%)	58 (70.7%)	159 (81.1%)	
IV	19 (13.9%)	6 (20.0%)	6 (15.4%)	19 (23.2%)	29 (14.8%)	
Histologic grade (*N* = 477)*						0.654
G2	19 (13.9%)	2 (6.9%)	6 (16.2%)	7 (8.8%)	23 (11.9%)	
G3	118 (86.1%)	27 (93.1%)	31 (83.8%)	73 (91.2%)	171 (88.1%)	
Residual tumor size (*N* = 432)*						0.213
No macroscopic disease	35 (28.0%)	9 (32.1%)	4 (12.5%)	10 (14.1%)	32 (18.2%)	
1–10 mm	61 (48.8%)	14 (50.0%)	22 (68.8%)	38 (53.5%)	88 (50.0%)	
11–20 mm	6 (4.8%)	2 (7.1%)	1 (3.1%)	7 (9.9%)	14 (7.9%)	
>20mm	23 (18.4%)	3 (10.7%)	5 (15.6%)	16 (22.5%)	42 (23.9%)	
Platinum response (*N* = 287)*						0.4
Resistant	24 (26.7%)	7 (41.2%)	5 (20.8%)	14 (31.1%)	40 (36.0%)	
Sensitive	66 (73.3%)	10 (58.8%)	19 (79.2%)	31 (68.9%)	71 (64.0%)	

*, sample size is different in each analysis

†, statistically significant

### Survival analysis and chemotherapy response

**Fig 3:**
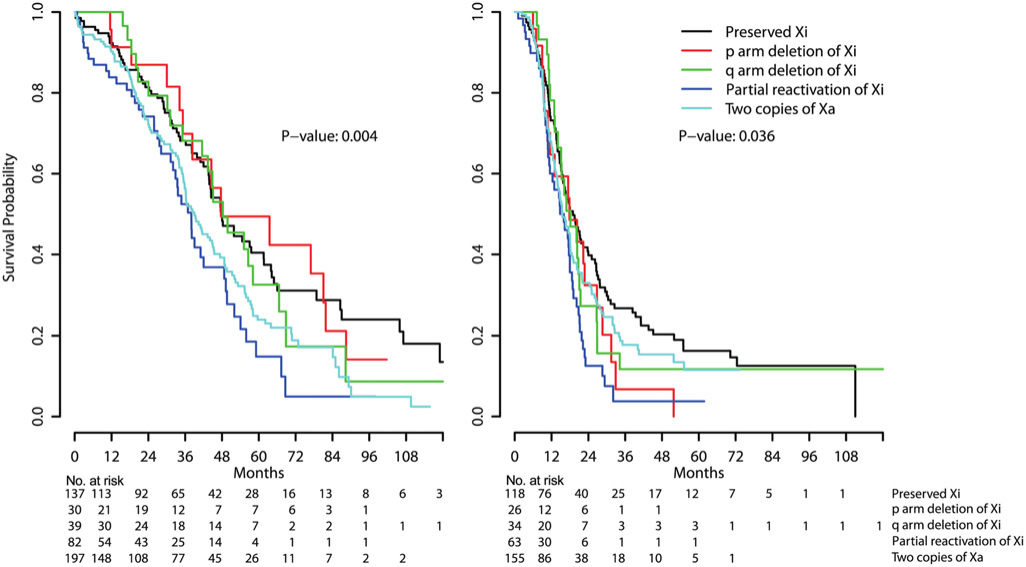
Prognosis of dysregulation of X chromosome inactivation. Overall (A) and progression free survival (B) were compared among groups of X chromosome inactivation status. The significances were estimated by overall log-rank test.

Of the 584 patients, 99 and 188 were excluded from the overall and progression free survival analysis, respectively, due to the absence of available data. Patients in the Xa^+^ and two Xa groups showed the worse overall survival (Fig. 3A). Patients in the Xa^+^ group showed worse progression free survival (Fig. 3B). The prognostic significance remained after adjusting for age, tumor stage, and residual tumor size using multivariate Cox regression test (Table 3). Patients in the Xi-p^-^ or Xi-q^-^ groups showed no survival differences when compared to the preserved Xi group. HG-SOA with Xi-p^-^, Xa^+^, or two Xa showed more frequently chemotherapy resistance, but this was not statistically significant (Table 2).

**Table 3:** Overall and progression free survival data using multivariate Cox regression test.

	Overall survival			Progression free survival		
	Median survival (95% CI)	HR (95% CI)	*P*-value	Median survival (95% CI)	HR (95% CI)	*P*-value
Age	NA	1.02 (1.00–1.03)	0.008*	NA	1.00 (0.99–1.01)	0.866
AJCC stage II	70.6 (47.5–NA)	Reference	Reference	26.8 (18.0–NA)	Reference	Reference
AJCC stage III	43.5 (39.1–47.7)	2.4 (0.9–6.0)	0.053	16.9 (15.4–18.9)	1.8 (0.9–3.3)	0.067
AJCC stage IV	33 (26.9–54.6)	2.8 (1.1–7.3)	0.029*	14 (11.5–18.1)	2.0 (1.0–4.1)	0.046*
No residual tumor	57.5 (47.5–NA)	Reference	Reference	21.6 (18.0–26.9)	Reference	Reference
Residual tumor size 1–10 mm	39.1 (36.2–45.0)	1.8 (1.2–2.6)	0.005*	15.1 (13.2–17.6)	1.6 (1.1–2.2)	0.011*
Residual tumor size 11–20 mm	39 (29.1–65.0)	1.6 (0.9–2.9)	0.088	13 (8.5–24.4)	1.6 (0.9–2.8)	0.0111
Residual tumor size >= 20 mm	34.8 (28.3–47.5)	1.7 (1.1–2.7)	0.019*	15.5 (14.1–19.5)	1.5 (0.9–2.2)	0.065
Preserved Xi	47.7 (43.8–61.7)	Reference	Reference	19 (15.6–25.1)	Reference	Reference
p arm deletion of Xi	47.7 (38.3–NA)	0.9 (0.5–1.6)	0.712	17.8 (11.5–31.5)	1.3 (0.8–2.2)	0.306
q arm deletion of Xi	48.3 (41.5–NA)	1.3 (0.8–2.2)	0.305	18.2 (15.1–26.7)	1.2 (0.7–2.0)	0.477
Partial reactivation of Xi	38 (32.1–49.2)	1.7 (1.1–2.5)	0.015*	16 (11.5–18.7)	1.7 (1.2–2.5)	0.007*
Two copies of Xa	39 (35.8–45.5)	1.4 (1.0–1.9)	0.030*	15.5 (14.0–18.2)	1.3 (0.9–1.7)	0.111

*, statistically significant

## Discussion

We found that dysregulation of XCI is common in HG-SOA and includes various different types of alterations, such as two copies of Xa, partial reactivation of Xi, and p or q arm deletion of Xi. Among those with dysregulation of XCI, two copies of Xa and partial reactivation of Xi increased the dose of Xa (loss of XCI). Loss of XCI was associated with poor prognosis in HG-SOA. Mutation or hypermethylation of *BRCA1* were infrequent in HG-SOAs with a loss of XCI. This result suggests that *BRCA1* dysfunction may not be related to the loss of XCI in HG-SOA.

Loss of XCI induced up-regulation of oncogenes located on the X chromosome was suggested as a mechanism of tumorigenesis in HG-SOA. However, it was unknown which gene was affected by the loss of XCI. We found four genes (*XAGE3*, *ZNF711*, *MAGEA4*, and *ZDHHC15*) that were up-regulated by loss of XCI. *XAGE3* and *MAGEA4* belong to a family of cancer-testis antigens. *MAGEA4* belongs to type 1 MAGEs, which cluster in the X chromosome and are regulated by methylation. *MAGEA4* was considered as a possible oncogene and a target for immunotherapy [25]. This result suggests that up-regulation of cancer-testis antigens has a role in the aggressiveness of HG-SOA with loss of XCI and explains the mechanism of aberrant expression of cancer-testis antigens in some HG-SOAs.

In previous study, most sporadic and *BRCA1*-associated, basal-like breast cancer showed two active X chromosomes and loss of XCI (61%), reactivation of XCI (22%), and gain of Xa (16%) [13]. This study also reported that genes located at Xp22 and Xq26–28 were over-expressed in basal-like breast cancer and suggested that over-expression of genes located at Xp22 was an important feature of basal-like breast cancer [13]. In our HG-SOA study, the preserved Xi group was more enriched higher expression of genes located at Xp22 than the two Xa group, contrary to the breast cancer study. The over-expression of genes located at Xp22 is a feature of basal-like breast cancer, but not HG-SOA with loss of XCI.

The Xa^+^ group showed more hypomethylation than the preserved Xi group, but less hypomethylation than the two Xa group. This represents partial reactivation of Xi. Global hypomethylation in cancer cells is known to occur. A previous report suggested that global hypomethylation is age dependent [26]. In HG-SOA, the Xa^+^ group showed more global hypomethylation and occurred in older patients. These results suggest that the partial reactivation of the X chromosome observed in the Xa^+^ group might be induced by global hypomethylation.

A previous study suggested that loss of XCI was associated with chemotherapy resistance in HG-SOA [27]. In our study, HG-SOA with a loss of XCI (Xa^+^ and two Xa) showed more chemotherapy resistance, but it was not statistically significant. The association between loss of XCI and chemotherapy response might be weak or absent. However, the small sample size might preclude a definite conclusion.

In our study, some HG-SOAs showed q arm deletions of Xi, which were associated with *BRCA1* germline mutations. The same q arm deletion of Xi has been reported in ovarian tumors with low malignant potential [4]. Two different pathways have recently been suggested for ovarian cancer and were designated ‘type I’ and ‘type II’ [28]. Type I ovarian serous cancer develops from benign serous adenoma and serous neoplasms of low malignant potential to low grade serous carcinoma. Type II develops from intraepithelial carcinomas of the fallopian tube or ovarian surface epithelium. HG-SOA is considered to be type II. HG-SOA with a q arm deletion of Xi suggests a different pathogenesis of HG-SOA associated with *BRCA1* germline mutation.

In conclusion, our observations provide evidence that dysregulation of XCI is common in HG-SOA and is associated with poor outcome. Loss of XCI induced up-regulation of cancer-testis antigens and this may have a role in tumor aggressiveness. The role of *BRCA1* in the loss of XCI might be limited. *BRCA1* germline mutations may have a role in the differential pathogenesis of HG-SOA associated with a q arm deletion of Xi.

## Supporting Information

S1 Fig *XIST* expression levels across different normal tissue. This data and figure is generated by The Genotype-Tissue Expression (GTEx) Consortium Analysis Working Group (http://www.gtexportal.org/home/). The Y-axis is reads per kilobase per million mapped reads (RPKM) of *XIST* and scaled logarithmically.
(TIF)

S2 Fig Genes regulated by loss of X chromosome inactivation. A) The points represent the more hypomethylated gene in the two Xa group than the preserved Xa group. X-axis is difference in mRNA expression z-scores between two Xa group and preserved Xi group. The difference of z-scores of mRNA and *P*-values were estimated using the Wilcox rank sum test. Four labeled genes are significantly more highly expressed in the two Xa group than in the preserved Xi group. B) Box plots represent the distribution of mRNA expression. The top and bottom of the box represent the 75^th^ and 25^th^ percentile, the line in the box represents median. The points represent the z-scores of mRNA expression.
(TIF)

S1 Table Post-hoc test of the difference in *XIST* expression. (DOCX)

S2 Table Chromosomal regions upregulated in two Xa group than preserved Xi group. (DOCX)

S3 Table Chromosomal regions upregulated in preserved Xi group than two Xa group. (DOCX)

S4 Table Post-hoc test of the difference in global methylation among clusters. (DOCX)

S5 Table Post-hoc test of age differences. (DOCX)

S1 Text Names of files downloaded from firehose website. (DOCX)
