# Supplementary figures and images for: Dysregulation of X Chromosome Inactivation in High Grade Ovarian Serous Adenocarcinoma
Source: PLoS One. 2015 Mar 5;10(3):e0118927. doi: 10.1371/journal.pone.0118927 (PMC4351149; doi:10.1371/journal.pone.0118927)

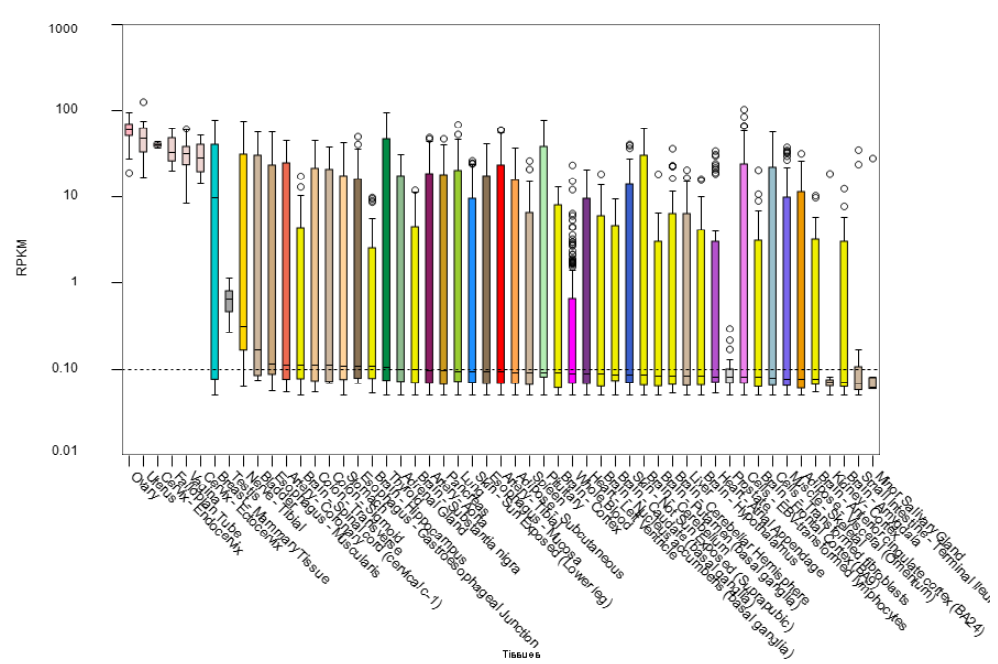

Supplement: S1 Fig — This data and figure is generated by The Genotype-Tissue Expression (GTEx) Consortium Analysis Working Group (http://www.gtexportal.org/home/). The Y-axis is reads per kilobase per million mapped reads (RPKM) of XIST and scaled logarithmically. (TIF) [file pone.0118927.s001.TIF]

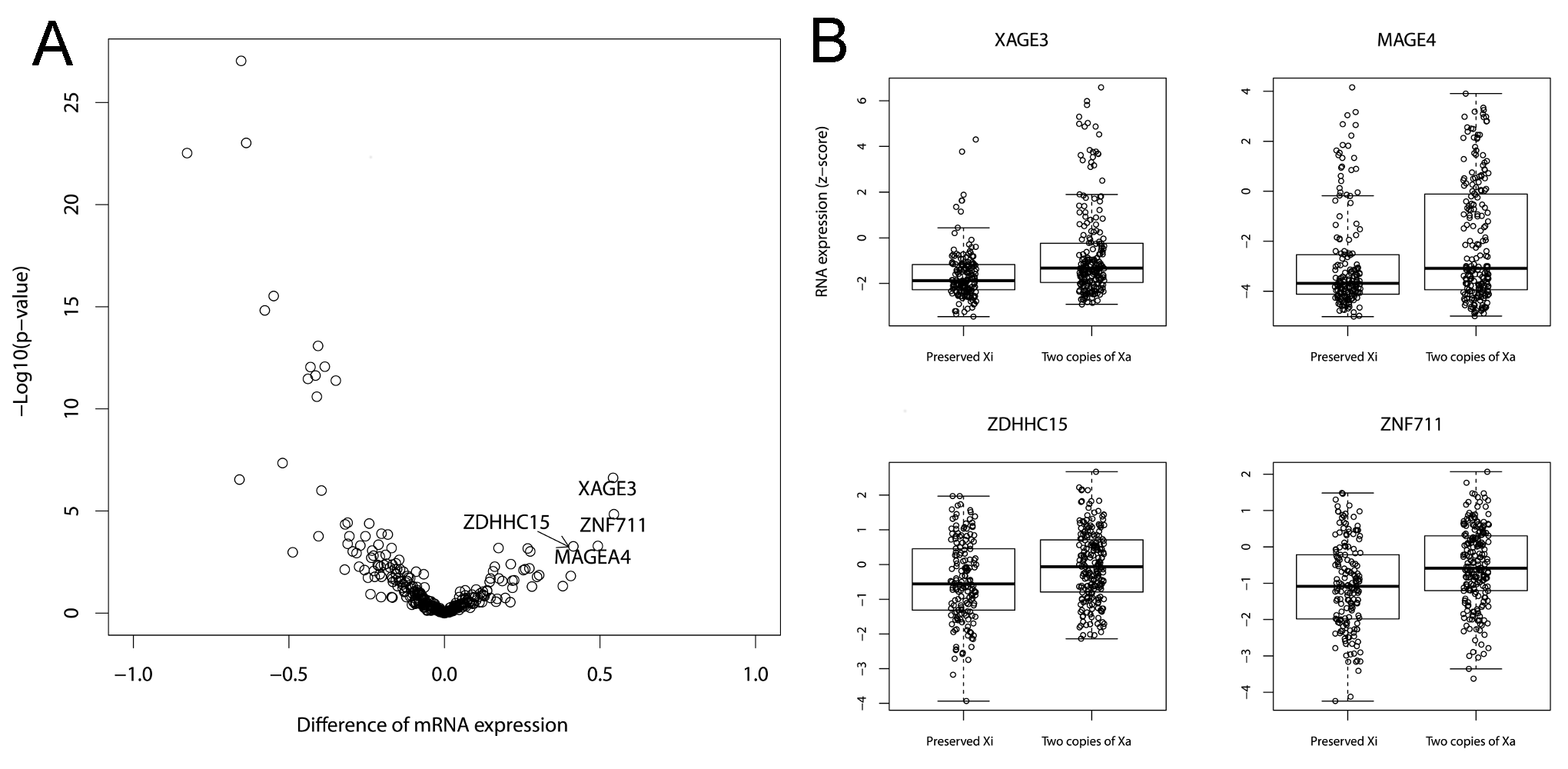

Supplement: S2 Fig — A) The points represent the more hypomethylated gene in the two Xa group than the preserved Xa group. X-axis is difference in mRNA expression z-scores between two Xa group and preserved Xi group. The difference of z-scores of mRNA and P-values were estimated using the Wilcox rank sum test. Four labeled genes are significantly more highly expressed in the two Xa group than in the preserved Xi group. B) Box plots represent the distribution of mRNA expression. The top and bottom of the box represent the 75th and 25th percentile, the line in the box represents median. The points represent the z-scores of mRNA expression. (TIF) [file pone.0118927.s002.tif]
