# Supplementary material for: Dysregulation of X Chromosome Inactivation in High Grade Ovarian Serous Adenocarcinoma
Source: PLoS One. 2015 Mar 5;10(3):e0118927. doi: 10.1371/journal.pone.0118927 (PMC4351149; doi:10.1371/journal.pone.0118927)
Supplement: S1 Table — (DOCX) [file pone.0118927.s003.docx]

Table S1: Post-hoc test of the difference in *XIST* expression among X chromosome methylation clusters using the TukeyHSD

| Cluster | Difference of the mean (95% CI) | Adjusted *P*-value |
| --- | --- | --- |
| 2-1 | -0.4(-0.9, 0.1) | 0.249 |
| 3-1 | -0.6 (-1.3, 0.1) | 0.16 |
| 4-1 | -0.9 (-1.5, -0.3) | <0.001 |
| 5-1 | -1.8 (-2.3, -1.4) | <0.001 |
| 6-1 | -2.7 (-3.2, -2.2) | <0.001 |
| 3-2 | -0.2 (-1.0, 0.6) | 0.98 |
| 4-2 | -0.5 (-1.2, 0.2) | 0.251 |
| 5-2 | -1.4 (-1.9, -0.9) | <0.001 |
| 6-2 | -2.3 (-2.8, -1.7) | <0.001 |
| 4-3 | -0.3 (-1.1, 0.5) | 0.877 |
| 5-3 | -1.2 (-1.9, -0.5) | <0.001 |
| 6-3 | -2.1 (-2.8, -1.3) | <0.001 |
| 5-4 | -0.9 (-1.5, -0.3) | <0.001 |
| 6-4 | -1.7 (-2.4, -1.1) | <0.001 |
| 6-5 | -0.8 (-1.3, -0.3) | <0.001 |
