# Supplementary material for: Dysregulation of X Chromosome Inactivation in High Grade Ovarian Serous Adenocarcinoma
Source: PLoS One. 2015 Mar 5;10(3):e0118927. doi: 10.1371/journal.pone.0118927 (PMC4351149; doi:10.1371/journal.pone.0118927)
Supplement: S2 Table — (DOCX) [file pone.0118927.s004.docx]

Table S2: Post-hoc test of the difference in global methylation among clusters using the TukeyHSD

| Cluster | Difference of the mean (95% CI) | Adjusted *P*-value |
| --- | --- | --- |
| 2-1 | -0.061 (-0.101, -0.021) | <0.001 |
| 3-1 | -0.035 (-0.094, 0.024) | 0.548 |
| 4-1 | 0.001 (-0.052, 0.055) | 0.999 |
| 5-1 | -0.039 (-0.076, -0.002) | 0.033 |
| 6-1 | -0.062 (-0.102, -0.022) | <0.001 |
| 3-2 | 0.026 (-0.037, 0.089) | 0.843 |
| 4-2 | 0.062 (0.004, 0.12) | 0.027 |
| 5-2 | 0.022 (-0.02, 0.064) | 0.674 |
| 6-2 | -0.001 (-0.047, 0.044) | 0.999 |
| 4-3 | 0.036 (-0.036, 0.108) | 0.711 |
| 5-3 | -0.004 (-0.065, 0.057) | 0.999 |
| 6-3 | -0.027 (-0.090, 0.036) | 0.819 |
| 5-4 | -0.04 (-0.096, 0.015) | 0.307 |
| 6-4 | -0.063 (-0.121, -0.005) | 0.023 |
| 6-5 | -0.023 (-0.066,0.019) | 0.630 |
