# Supplementary material for: Dysregulation of X Chromosome Inactivation in High Grade Ovarian Serous Adenocarcinoma
Source: PLoS One. 2015 Mar 5;10(3):e0118927. doi: 10.1371/journal.pone.0118927 (PMC4351149; doi:10.1371/journal.pone.0118927)
Supplement: S3 Table — (DOCX) [file pone.0118927.s005.docx]

Table S3: Post-hoc test of age differences among X chromosome inactivation status groups using the TukeyHSD

| Group | Difference of the mean (95% CI) | Adjusted *P*-value |
| --- | --- | --- |
| p arm deletion of Xi-Preserved Xi | 0.5 (-5.3, 6.3) | 0.999 |
| q arm deletion of Xi-Preserved Xi | -2.7 (-8.0, 2.6) | 0.634 |
| Partial reactivation of Xi-Preserved Xi | 4.6 (0.6, 8.6) | 0.014 |
| Two copies of Xa-Preserved Xi | 3.9 (0.7, 7.0) | 0.008 |
| q arm deletion of Xi-p arm deletion of Xi | -3.2 (-10.3, 3.9) | 0.736 |
| Partial reactivation of Xi-p arm deletion of Xi | 4.1 (-2.1, 10.3) | 0.362 |
| Two copies of Xa-p arm deletion of Xi | 3.4 (-2.3, 9.1) | 0.477 |
| Partial reactivation of Xi-q arm deletion of Xi | 7.3 (1.6, 13) | 0.005 |
| Two copies of Xa-q arm deletion of Xi | 6.6 (1.4, 11.8) | 0.005 |
| Two copies of Xa-partial reactivation of Xi | -0.7 (-4.5, 3.1) | 0.986 |
