# Supplementary material for: Dysregulation of X Chromosome Inactivation in High Grade Ovarian Serous Adenocarcinoma
Source: PLoS One. 2015 Mar 5;10(3):e0118927. doi: 10.1371/journal.pone.0118927 (PMC4351149; doi:10.1371/journal.pone.0118927)
Supplement: S4 Table — (DOCX) [file pone.0118927.s006.docx]

Table S5. Gene sets (chromosomal region) are upregulated in two Xa group than preserved Xi group

| Region | Number of genes | Enrichment Score (ES) | Normalized Enrichment Score (NES) | Nominal p-value | FDR q-value | FWER p-Value |
| --- | --- | --- | --- | --- | --- | --- |
| CHR20Q11 | 83 | -0.64443 | -1.91283 | <0.001 | 0.755852 | 0.419 |
| CHR3Q29 | 36 | -0.66877 | -1.88757 | 0.005848 | 0.480895 | 0.498 |
| CHR8Q11 | 20 | -0.64825 | -1.7467 | 0.02834 | 1 | 0.883 |
| CHR20P11 | 41 | -0.60138 | -1.72521 | 0.035294 | 0.907055 | 0.913 |
| CHR1P36 | 269 | -0.50276 | -1.71686 | 0.041176 | 0.770434 | 0.924 |
| CHR12P11 | 29 | -0.60317 | -1.6198 | 0.040241 | 1 | 0.987 |
| CHR20P12 | 33 | -0.59962 | -1.61925 | 0.056751 | 1 | 0.988 |
| CHR17P13 | 182 | -0.4969 | -1.59133 | 0.059305 | 1 | 0.995 |
| CHR12P12 | 49 | -0.51525 | -1.57086 | 0.070881 | 1 | 0.996 |
| CHR14Q22 | 42 | -0.57366 | -1.55656 | 0.107595 | 0.989039 | 0.997 |
| CHR3Q28 | 16 | -0.62341 | -1.54059 | 0.068548 | 0.977506 | 0.998 |
| CHR3Q27 | 48 | -0.52317 | -1.53945 | 0.070686 | 0.901032 | 0.999 |
| CHR12Q23 | 54 | -0.51296 | -1.5324 | 0.09165 | 0.859529 | 0.999 |
| CHR8Q12 | 21 | -0.57919 | -1.52162 | 0.073171 | 0.8392 | 0.999 |
| CHR1Q41 | 28 | -0.53451 | -1.50193 | 0.079268 | 0.861931 | 0.999 |
| CHR3Q12 | 25 | -0.56569 | -1.48821 | 0.11306 | 0.860756 | 1 |
| CHR10P13 | 20 | -0.5855 | -1.48592 | 0.090211 | 0.818312 | 1 |
| CHR1Q44 | 39 | -0.49902 | -1.47044 | 0.09002 | 0.826788 | 1 |
| CHR12P13 | 158 | -0.42241 | -1.46546 | 0.104167 | 0.79931 | 1 |
| CHR17Q22 | 27 | -0.49573 | -1.46491 | 0.105809 | 0.760934 | 1 |
| CHR17Q11 | 91 | -0.42701 | -1.46461 | 0.086519 | 0.726012 | 1 |
| CHR5Q13 | 49 | -0.50318 | -1.44816 | 0.140206 | 0.741076 | 1 |
| CHR13Q14 | 63 | -0.50167 | -1.44418 | 0.157233 | 0.719035 | 1 |
| CHR10P11 | 25 | -0.51911 | -1.43992 | 0.105973 | 0.701418 | 1 |
| CHR18P11 | 51 | -0.48172 | -1.43058 | 0.156313 | 0.698987 | 1 |
| CHR3Q26 | 46 | -0.47511 | -1.42891 | 0.140244 | 0.676569 | 1 |
| CHR10P12 | 32 | -0.48478 | -1.38599 | 0.149425 | 0.767643 | 1 |
| CHR10Q22 | 77 | -0.42982 | -1.38496 | 0.154599 | 0.743136 | 1 |
| CHR1Q21 | 213 | -0.37523 | -1.37816 | 0.127202 | 0.736612 | 1 |
| CHR18Q11 | 28 | -0.51685 | -1.36458 | 0.163386 | 0.74785 | 1 |
| CHR3Q21 | 89 | -0.41288 | -1.30969 | 0.180328 | 0.876561 | 1 |
| CHR1P35 | 75 | -0.43518 | -1.30896 | 0.22736 | 0.851311 | 1 |
| CHR20P13 | 60 | -0.44178 | -1.3027 | 0.209615 | 0.84405 | 1 |
| CHR2P16 | 24 | -0.48715 | -1.29799 | 0.197652 | 0.832485 | 1 |
| CHR1Q42 | 81 | -0.44154 | -1.27965 | 0.24165 | 0.857 | 1 |
| CHR10Q26 | 78 | -0.39073 | -1.26649 | 0.217391 | 0.871123 | 1 |
| CHR5Q14 | 32 | -0.47477 | -1.26075 | 0.247899 | 0.861666 | 1 |
| CHR12Q22 | 24 | -0.47455 | -1.25531 | 0.255814 | 0.854476 | 1 |
| CHR2P11 | 38 | -0.41506 | -1.25458 | 0.24187 | 0.83446 | 1 |
| CHR6P24 | 22 | -0.47916 | -1.24928 | 0.244 | 0.827413 | 1 |
| CHR14Q24 | 80 | -0.40706 | -1.2438 | 0.267068 | 0.820246 | 1 |
| CHR5Q21 | 20 | -0.47662 | -1.2435 | 0.263052 | 0.801214 | 1 |
| CHR2Q23 | 18 | -0.47229 | -1.2315 | 0.2625 | 0.81072 | 1 |
| CHR10Q25 | 38 | -0.4126 | -1.22784 | 0.247485 | 0.801226 | 1 |
| CHR17Q21 | 225 | -0.33562 | -1.22254 | 0.233684 | 0.795292 | 1 |
| CHR3Q13 | 71 | -0.36689 | -1.20956 | 0.251586 | 0.80792 | 1 |
| CHR17P11 | 57 | -0.4002 | -1.20452 | 0.259958 | 0.802537 | 1 |
| CHR2P24 | 40 | -0.40464 | -1.1882 | 0.283433 | 0.821852 | 1 |
| CHR1P32 | 69 | -0.37932 | -1.18718 | 0.295775 | 0.807305 | 1 |
| CHR17P12 | 23 | -0.40485 | -1.17707 | 0.261856 | 0.814034 | 1 |
| CHR2Q13 | 23 | -0.42011 | -1.16117 | 0.319756 | 0.834323 | 1 |
| CHR10P14 | 15 | -0.47432 | -1.1607 | 0.330769 | 0.819785 | 1 |
| CHR5Q12 | 29 | -0.44153 | -1.15982 | 0.323651 | 0.806646 | 1 |
| CHR10Q24 | 91 | -0.37435 | -1.14069 | 0.341897 | 0.832235 | 1 |
| CHR1Q22 | 63 | -0.34568 | -1.13876 | 0.278311 | 0.820975 | 1 |
| CHR2P23 | 61 | -0.36831 | -1.13041 | 0.338462 | 0.824525 | 1 |
| CHR1Q43 | 16 | -0.43755 | -1.10928 | 0.355556 | 0.855197 | 1 |
| CHR19P12 | 26 | -0.4781 | -1.10875 | 0.38755 | 0.841411 | 1 |
| CHR3Q22 | 32 | -0.41283 | -1.09931 | 0.367886 | 0.84635 | 1 |
| CHR5Q15 | 15 | -0.41966 | -1.09614 | 0.34874 | 0.838582 | 1 |
| CHR11P14 | 20 | -0.38846 | -1.07924 | 0.357977 | 0.858859 | 1 |
| CHR5Q11 | 29 | -0.40674 | -1.07624 | 0.404472 | 0.851115 | 1 |
| CHR6Q14 | 30 | -0.38326 | -1.07571 | 0.39749 | 0.838834 | 1 |
| CHR10Q21 | 32 | -0.35824 | -1.0531 | 0.419422 | 0.871323 | 1 |
| CHR12Q24 | 177 | -0.3397 | -1.05179 | 0.396414 | 0.8605 | 1 |
| CHR6P22 | 88 | -0.36045 | -1.03847 | 0.43254 | 0.87321 | 1 |
| CHR12Q15 | 22 | -0.36997 | -1.03801 | 0.401247 | 0.860921 | 1 |
| CHR1P34 | 131 | -0.34434 | -1.03619 | 0.413927 | 0.851708 | 1 |
| CHR14Q31 | 20 | -0.39761 | -1.03175 | 0.448133 | 0.847771 | 1 |
| CHR8P11 | 39 | -0.35516 | -1.02317 | 0.445973 | 0.851478 | 1 |
| CHR20Q13 | 155 | -0.30175 | -1.01335 | 0.405253 | 0.857695 | 1 |
| CHR2P22 | 36 | -0.36545 | -1.00887 | 0.464 | 0.854325 | 1 |
| CHR7Q22 | 83 | -0.32941 | -1.00637 | 0.457854 | 0.846775 | 1 |
| CHR2P21 | 33 | -0.35972 | -1.00342 | 0.45122 | 0.840376 | 1 |
| CHR7Q21 | 69 | -0.29819 | -0.99205 | 0.446184 | 0.849119 | 1 |
| CHR5Q35 | 82 | -0.34988 | -0.98302 | 0.486111 | 0.854392 | 1 |
| CHR10Q23 | 74 | -0.30995 | -0.97821 | 0.468619 | 0.851949 | 1 |
| CHR14Q13 | 24 | -0.36658 | -0.97804 | 0.481633 | 0.841283 | 1 |
| CHR2P25 | 45 | -0.34915 | -0.9732 | 0.46988 | 0.83929 | 1 |
| CHR1Q25 | 55 | -0.3069 | -0.95372 | 0.503861 | 0.863215 | 1 |
| CHR14Q21 | 32 | -0.33393 | -0.95032 | 0.493878 | 0.858916 | 1 |
| CHR20Q12 | 36 | -0.30355 | -0.94797 | 0.505906 | 0.852453 | 1 |
| CHR4Q32 | 24 | -0.3435 | -0.94562 | 0.538767 | 0.846185 | 1 |
| CHR8Q22 | 58 | -0.33958 | -0.93703 | 0.514228 | 0.850831 | 1 |
| CHR11Q21 | 26 | -0.35226 | -0.93689 | 0.556452 | 0.840971 | 1 |
| CHR1P31 | 69 | -0.29527 | -0.93627 | 0.507099 | 0.832328 | 1 |
| CHR18Q12 | 37 | -0.31677 | -0.93187 | 0.518738 | 0.830326 | 1 |
| CHR5P13 | 41 | -0.31331 | -0.92996 | 0.524793 | 0.823808 | 1 |
| CHR4Q13 | 45 | -0.28902 | -0.92303 | 0.542443 | 0.82572 | 1 |
| CHR2Q33 | 71 | -0.29121 | -0.91812 | 0.539924 | 0.82473 | 1 |
| CHR1Q24 | 36 | -0.30929 | -0.91353 | 0.514395 | 0.823224 | 1 |
| CHR14Q23 | 34 | -0.33077 | -0.90722 | 0.528067 | 0.824911 | 1 |
| CHR12Q | 15 | -0.34113 | -0.90031 | 0.572614 | 0.827051 | 1 |
| CHR11Q25 | 15 | -0.35188 | -0.88856 | 0.578288 | 0.836578 | 1 |
| CHR10P15 | 34 | -0.30363 | -0.87973 | 0.586481 | 0.841156 | 1 |
| CHR5Q33 | 43 | -0.29362 | -0.87171 | 0.608696 | 0.84494 | 1 |
| CHR2Q24 | 35 | -0.29147 | -0.87074 | 0.607595 | 0.837814 | 1 |
| CHR3Q23 | 22 | -0.3232 | -0.86655 | 0.584615 | 0.835572 | 1 |
| CHR2P13 | 62 | -0.26473 | -0.86329 | 0.601167 | 0.832415 | 1 |
| CHR5P15 | 57 | -0.28678 | -0.85967 | 0.596806 | 0.830076 | 1 |
| CHR6Q21 | 46 | -0.31355 | -0.84354 | 0.6 | 0.844879 | 1 |
| CHR13Q12 | 63 | -0.29014 | -0.84203 | 0.592593 | 0.838998 | 1 |
| CHR12Q14 | 33 | -0.30379 | -0.83794 | 0.60241 | 0.837068 | 1 |
| CHR1Q32 | 118 | -0.24264 | -0.83432 | 0.637965 | 0.834804 | 1 |
| CHR8P12 | 26 | -0.28917 | -0.83003 | 0.632411 | 0.83313 | 1 |
| CHR12Q21 | 33 | -0.28642 | -0.82429 | 0.627368 | 0.83338 | 1 |
| CHR7P13 | 30 | -0.31155 | -0.82164 | 0.614931 | 0.829641 | 1 |
| CHR3Q25 | 43 | -0.28804 | -0.82136 | 0.654076 | 0.822423 | 1 |
| CHR4Q12 | 29 | -0.28883 | -0.81795 | 0.628631 | 0.819323 | 1 |
| CHR4P12 | 18 | -0.29889 | -0.81736 | 0.678862 | 0.812614 | 1 |
| CHR17Q23 | 54 | -0.25892 | -0.79498 | 0.696787 | 0.836767 | 1 |
| CHR1P33 | 31 | -0.26932 | -0.79241 | 0.718447 | 0.833058 | 1 |
| CHR2Q12 | 35 | -0.25957 | -0.78816 | 0.730539 | 0.831716 | 1 |
| CHR2Q11 | 44 | -0.24392 | -0.7832 | 0.689516 | 0.831245 | 1 |
| CHR4Q25 | 30 | -0.27428 | -0.76904 | 0.711864 | 0.842199 | 1 |
| CHR13Q13 | 23 | -0.30327 | -0.76317 | 0.679834 | 0.84339 | 1 |
| CHR2P12 | 23 | -0.25766 | -0.74824 | 0.784158 | 0.856721 | 1 |
| CHR2Q32 | 37 | -0.26353 | -0.74365 | 0.729839 | 0.855653 | 1 |
| CHR5Q23 | 47 | -0.23717 | -0.71656 | 0.750515 | 0.884099 | 1 |
| CHR5Q34 | 23 | -0.25172 | -0.7163 | 0.795229 | 0.877149 | 1 |
| CHR17Q24 | 31 | -0.25052 | -0.70077 | 0.801688 | 0.890368 | 1 |
| CHR8Q23 | 22 | -0.2541 | -0.68628 | 0.818363 | 0.902558 | 1 |
| CHR2P14 | 24 | -0.22705 | -0.64678 | 0.850895 | 0.944054 | 1 |
| CHR6Q22 | 48 | -0.22637 | -0.63225 | 0.817073 | 0.954644 | 1 |
| CHR11P15 | 249 | -0.17067 | -0.63176 | 0.908024 | 0.947636 | 1 |
| CHR3P24 | 32 | -0.2312 | -0.63053 | 0.856557 | 0.94143 | 1 |
| CHR7Q11 | 71 | -0.20172 | -0.62332 | 0.863281 | 0.942251 | 1 |
| CHR12Q12 | 54 | -0.18657 | -0.61831 | 0.920792 | 0.940288 | 1 |
| CHR8P21 | 71 | -0.19997 | -0.59231 | 0.862275 | 0.96112 | 1 |
| CHR7Q32 | 31 | -0.20341 | -0.58419 | 0.92543 | 0.962057 | 1 |
| CHR7P14 | 36 | -0.19042 | -0.56038 | 0.920082 | 0.976819 | 1 |
| CHR12Q13 | 188 | -0.16499 | -0.55964 | 0.960474 | 0.970044 | 1 |
| CHR6Q25 | 43 | -0.17963 | -0.51291 | 0.950515 | 0.997431 | 1 |
| CHR18Q21 | 63 | -0.17116 | -0.50663 | 0.941532 | 0.993959 | 1 |
| CHR4Q31 | 53 | -0.15513 | -0.43696 | 0.971717 | 1 | 1 |
| CHR6Q16 | 18 | -0.16457 | -0.42305 | 0.995833 | 1 | 1 |
| CHR8Q13 | 32 | -0.14068 | -0.39015 | 1 | 1 | 1 |
| CHR4P14 | 23 | -0.13255 | -0.35714 | 0.993724 | 1 | 1 |
| CHR1P21 | 28 | -0.1247 | -0.34075 | 0.994129 | 0.998964 | 1 |
