# Supplementary material for: Dysregulation of X Chromosome Inactivation in High Grade Ovarian Serous Adenocarcinoma
Source: PLoS One. 2015 Mar 5;10(3):e0118927. doi: 10.1371/journal.pone.0118927 (PMC4351149; doi:10.1371/journal.pone.0118927)
Supplement: S5 Table — (DOCX) [file pone.0118927.s007.docx]

Table S4. Gene sets (chromosomal region) are upregulated in preserved Xi group than two Xa group

| Region | Number of genes | Enrichment Score (ES) | Normalized Enrichment Score (NES) | Nominal p-value | FDR q-value | FWER p-Value |
| --- | --- | --- | --- | --- | --- | --- |
| CHR9Q22 | 68 | 0.70993 | 1.9914931 | 0.001960784 | 0.24669729 | 0.223 |
| CHRXP22 | 92 | 0.61813945 | 1.9445924 | 0.011695907 | 0.21067551 | 0.333 |
| CHR9Q32 | 30 | 0.6656636 | 1.8510644 | 0.011741683 | 0.35838583 | 0.626 |
| CHR9P24 | 38 | 0.6659485 | 1.8160034 | 0.023060797 | 0.36480904 | 0.733 |
| CHR16Q23 | 24 | 0.65461874 | 1.7764972 | 0.022 | 0.39784476 | 0.825 |
| CHR9Q31 | 44 | 0.5841293 | 1.7224765 | 0.03696498 | 0.49476558 | 0.925 |
| CHR15Q13 | 23 | 0.65393907 | 1.707501 | 0.031558186 | 0.46726596 | 0.947 |
| CHR9Q34 | 192 | 0.56826705 | 1.6960113 | 0.04660194 | 0.4429696 | 0.959 |
| CHR16Q24 | 65 | 0.560619 | 1.636475 | 0.07172131 | 0.5722762 | 0.992 |
| CHR16Q22 | 113 | 0.5426661 | 1.6254119 | 0.08097166 | 0.5478638 | 0.994 |
| CHR15Q15 | 56 | 0.54946744 | 1.6188029 | 0.0734127 | 0.51580095 | 0.994 |
| CHR9Q33 | 54 | 0.513566 | 1.6065927 | 0.044573642 | 0.5055969 | 0.996 |
| CHR16P12 | 63 | 0.49923036 | 1.536209 | 0.08316832 | 0.67079186 | 0.999 |
| CHR9Q21 | 42 | 0.5275864 | 1.4926645 | 0.10754717 | 0.76328903 | 1 |
| CHRXQ25 | 18 | 0.55330616 | 1.46339 | 0.0917603 | 0.80731916 | 1 |
| CHR16Q21 | 16 | 0.5964324 | 1.4533693 | 0.115234375 | 0.7884013 | 1 |
| CHR15Q23 | 26 | 0.5231626 | 1.4498085 | 0.14450867 | 0.7529388 | 1 |
| CHR9P21 | 25 | 0.54854023 | 1.4159493 | 0.14884695 | 0.81487334 | 1 |
| CHRXQ24 | 28 | 0.5261312 | 1.4066223 | 0.1509434 | 0.80107975 | 1 |
| CHR11Q13 | 217 | 0.4205688 | 1.3853649 | 0.17322835 | 0.827648 | 1 |
| CHR9P13 | 45 | 0.47450337 | 1.3706907 | 0.17083333 | 0.8325818 | 1 |
| CHR11P11 | 46 | 0.43422094 | 1.3560785 | 0.15936255 | 0.8389324 | 1 |
| CHR15Q21 | 72 | 0.4361554 | 1.3347207 | 0.21235521 | 0.86242497 | 1 |
| CHRXQ27 | 17 | 0.4974953 | 1.3108166 | 0.19361277 | 0.89387506 | 1 |
| CHR15Q14 | 34 | 0.46368057 | 1.2966139 | 0.20682731 | 0.89856607 | 1 |
| CHR22Q11 | 101 | 0.4367962 | 1.294031 | 0.22330096 | 0.87125784 | 1 |
| CHR19P13 | 440 | 0.38537738 | 1.2938517 | 0.20654397 | 0.8396623 | 1 |
| CHR16Q13 | 36 | 0.46439 | 1.2693044 | 0.23517382 | 0.8724534 | 1 |
| CHR16P11 | 77 | 0.42559716 | 1.269103 | 0.2570281 | 0.8428452 | 1 |
| CHR7Q34 | 41 | 0.4026554 | 1.2321836 | 0.23123732 | 0.9139287 | 1 |
| CHR3P22 | 44 | 0.43001002 | 1.2284431 | 0.26185566 | 0.8937318 | 1 |
| CHR15Q11 | 27 | 0.42133558 | 1.2280148 | 0.24609375 | 0.86696863 | 1 |
| CHR22Q13 | 144 | 0.3883332 | 1.2159965 | 0.27327934 | 0.8713244 | 1 |
| CHRXQ13 | 48 | 0.3803453 | 1.20079 | 0.2593985 | 0.8825043 | 1 |
| CHR11Q22 | 45 | 0.3991111 | 1.1619024 | 0.31226054 | 0.95390964 | 1 |
| CHRXQ28 | 84 | 0.38120145 | 1.1605068 | 0.32553607 | 0.93063426 | 1 |
| CHR15Q22 | 66 | 0.4073999 | 1.1522484 | 0.34560326 | 0.9252922 | 1 |
| CHR16Q12 | 33 | 0.44433314 | 1.1517621 | 0.3503937 | 0.9022914 | 1 |
| CHR14Q12 | 19 | 0.4222011 | 1.1186544 | 0.35104364 | 0.95504504 | 1 |
| CHR2Q35 | 44 | 0.3615956 | 1.1035041 | 0.34086242 | 0.9659973 | 1 |
| CHR8P23 | 40 | 0.36577573 | 1.0879245 | 0.3618677 | 0.97769177 | 1 |
| CHR3P25 | 64 | 0.36706227 | 1.0769223 | 0.3979798 | 0.9797752 | 1 |
| CHR13Q32 | 25 | 0.38642088 | 1.0746006 | 0.40319362 | 0.96194965 | 1 |
| CHR9P22 | 29 | 0.36816782 | 1.0652614 | 0.3677686 | 0.9604063 | 1 |
| CHRXP21 | 28 | 0.33558267 | 1.0359137 | 0.40041068 | 1 | 1 |
| CHR3P21 | 202 | 0.31244755 | 1.0168037 | 0.45726496 | 1 | 1 |
| CHR11Q11 | 51 | 0.30670205 | 1.0084064 | 0.45816734 | 1 | 1 |
| CHR16P13 | 198 | 0.3259561 | 0.99955934 | 0.45309383 | 1 | 1 |
| CHR6P23 | 16 | 0.41259483 | 0.9857496 | 0.46825397 | 1 | 1 |
| CHR3P26 | 19 | 0.3891957 | 0.9776272 | 0.50715744 | 1 | 1 |
| CHR15Q24 | 57 | 0.32554084 | 0.97314495 | 0.48565573 | 1 | 1 |
| CHR15Q25 | 37 | 0.34962425 | 0.9674258 | 0.48846152 | 1 | 1 |
| CHR11Q23 | 103 | 0.30369952 | 0.962849 | 0.509542 | 1 | 1 |
| CHR4Q23 | 17 | 0.38553208 | 0.9402892 | 0.54022986 | 1 | 1 |
| CHR2Q37 | 83 | 0.29839796 | 0.93702376 | 0.52556235 | 1 | 1 |
| CHR11Q12 | 118 | 0.26025045 | 0.9286396 | 0.5510597 | 1 | 1 |
| CHR11Q24 | 58 | 0.28201738 | 0.91734344 | 0.53169733 | 1 | 1 |
| CHR4Q28 | 28 | 0.3204632 | 0.90224564 | 0.54545456 | 1 | 1 |
| CHR8Q24 | 118 | 0.3223434 | 0.8928754 | 0.57114226 | 1 | 1 |
| CHRXQ22 | 65 | 0.29940158 | 0.8905907 | 0.55212355 | 1 | 1 |
| CHR4Q35 | 28 | 0.31892726 | 0.88959247 | 0.57086617 | 1 | 1 |
| CHR7Q36 | 56 | 0.30404758 | 0.8863258 | 0.5743381 | 0.9955036 | 1 |
| CHR13Q34 | 28 | 0.34986934 | 0.8762201 | 0.5947581 | 0.99902153 | 1 |
| CHR21Q22 | 140 | 0.24964836 | 0.8505903 | 0.6184486 | 1 | 1 |
| CHR10Q11 | 50 | 0.25487792 | 0.8490947 | 0.62618595 | 1 | 1 |
| CHR11P13 | 34 | 0.300328 | 0.8459871 | 0.60162604 | 1 | 1 |
| CHR1Q31 | 39 | 0.25467247 | 0.84525657 | 0.6614173 | 0.9942725 | 1 |
| CHR7P15 | 56 | 0.2741657 | 0.8285417 | 0.61507934 | 1 | 1 |
| CHR5Q32 | 27 | 0.28110236 | 0.81541926 | 0.66805845 | 1 | 1 |
| CHR3P14 | 33 | 0.2722309 | 0.8094927 | 0.68810916 | 1 | 1 |
| CHR7Q35 | 27 | 0.28148472 | 0.7967465 | 0.6783505 | 1 | 1 |
| CHR1Q23 | 75 | 0.22207883 | 0.7877729 | 0.741483 | 1 | 1 |
| CHR1P22 | 65 | 0.26874214 | 0.78519964 | 0.6780684 | 1 | 1 |
| CHR8P22 | 34 | 0.26345783 | 0.7763525 | 0.7209776 | 1 | 1 |
| CHR4Q22 | 30 | 0.25973308 | 0.7736326 | 0.709434 | 1 | 1 |
| CHR2Q36 | 35 | 0.23279011 | 0.7636857 | 0.76374745 | 1 | 1 |
| CHRXP11 | 127 | 0.23548608 | 0.7611157 | 0.68875504 | 0.9967684 | 1 |
| CHR14Q32 | 99 | 0.23817717 | 0.73751205 | 0.7106796 | 1 | 1 |
| CHR6P25 | 31 | 0.26220155 | 0.7348898 | 0.7455969 | 1 | 1 |
| CHR2Q14 | 30 | 0.24279702 | 0.73059374 | 0.7809524 | 1 | 1 |
| CHRXQ26 | 41 | 0.22994448 | 0.726099 | 0.7805343 | 1 | 1 |
| CHR5Q22 | 20 | 0.30526027 | 0.71664387 | 0.7351779 | 1 | 1 |
| CHR6P12 | 45 | 0.2287604 | 0.7107389 | 0.794 | 1 | 1 |
| CHR22Q12 | 92 | 0.23529392 | 0.7024834 | 0.75298804 | 1 | 1 |
| CHR8Q21 | 47 | 0.23311152 | 0.69598913 | 0.79116464 | 0.998808 | 1 |
| CHR6P21 | 283 | 0.205476 | 0.6951145 | 0.7768924 | 0.98844206 | 1 |
| CHRXQ21 | 35 | 0.23428018 | 0.6916976 | 0.83820665 | 0.9821144 | 1 |
| CHR15Q26 | 51 | 0.24372542 | 0.6909692 | 0.75151515 | 0.97208655 | 1 |
| CHR7P21 | 31 | 0.23091763 | 0.6719869 | 0.7916667 | 0.98733824 | 1 |
| CHR1P13 | 90 | 0.20128687 | 0.6719528 | 0.84126985 | 0.97641826 | 1 |
| CHR18Q22 | 21 | 0.2521388 | 0.66213214 | 0.82738096 | 0.97841984 | 1 |
| CHR4P16 | 87 | 0.21788044 | 0.65459424 | 0.792 | 0.97799253 | 1 |
| CHR4Q21 | 72 | 0.19843791 | 0.62805796 | 0.87145555 | 1 | 1 |
| CHR6Q23 | 39 | 0.20921029 | 0.6277015 | 0.9011194 | 0.9912968 | 1 |
| CHR7Q31 | 74 | 0.17769502 | 0.6177352 | 0.9327902 | 0.9922964 | 1 |
| CHRYQ11 | 19 | 0.24477334 | 0.6086855 | 0.8709056 | 0.99270326 | 1 |
| CHR2Q21 | 36 | 0.19923161 | 0.59845185 | 0.8993711 | 0.9945951 | 1 |
| CHR17Q25 | 144 | 0.20509088 | 0.5970607 | 0.83168316 | 0.9860327 | 1 |
| CHR2Q31 | 64 | 0.19469124 | 0.59583026 | 0.88777554 | 0.97759736 | 1 |
| CHR3Q24 | 18 | 0.21673405 | 0.5946934 | 0.9192913 | 0.9688402 | 1 |
| CHR6Q24 | 38 | 0.20932515 | 0.5779379 | 0.8657845 | 0.97603697 | 1 |
| CHR7Q33 | 19 | 0.18972637 | 0.5541416 | 0.9758551 | 0.98790395 | 1 |
| CHR5Q31 | 159 | 0.16109678 | 0.530832 | 0.9452333 | 0.9977498 | 1 |
| CHR4P15 | 32 | 0.19482021 | 0.530735 | 0.9253438 | 0.98821455 | 1 |
| CHR17Q12 | 60 | 0.14724678 | 0.49939874 | 0.9903101 | 0.9997774 | 1 |
| CHR14Q11 | 114 | 0.14645974 | 0.4889313 | 0.98015875 | 0.99535865 | 1 |
| CHR6Q27 | 28 | 0.1709826 | 0.4686959 | 0.9716599 | 0.9952395 | 1 |
| CHR7P22 | 53 | 0.16936713 | 0.4383447 | 0.9485149 | 0.996403 | 1 |
| CHR11Q14 | 35 | 0.14764474 | 0.419669 | 0.99233717 | 0.9914412 | 1 |
