# Supplementary material for: Dysregulation of X Chromosome Inactivation in High Grade Ovarian Serous Adenocarcinoma
Source: PLoS One. 2015 Mar 5;10(3):e0118927. doi: 10.1371/journal.pone.0118927 (PMC4351149; doi:10.1371/journal.pone.0118927)
Supplement: S1 Text — (DOCX) [file pone.0118927.s008.docx]

**Text S1. Names of files downloaded from firehose website**

1. DNA methylation: gdac.broadinstitute.org_OV.Merge_methylation__humanmethylation27__jhu_usc_edu__Level_3__within_bioassay_data_set_function__data.Level_3.2014051800.0.0.tar

2. Segmented copy number:

gdac.broadinstitute.org_OV.Merge_snp__genome_wide_snp_6__broad_mit_edu__Level_3__segmented_scna_minus_germline_cnv_hg19__seg.Level_3.2014071500.0.0.tar.gz

3. Segmental loss of heterozygosity:

gdac.broadinstitute.org_OV.Merge_snp__human1mduo__hudsonalpha_org__Level_3__segmented_loh__seg.Level_3.2014090200.0.0.tar

4. RNA-seq:

gdac.broadinstitute.org_OV.Merge_rnaseqv2__illuminahiseq_rnaseqv2__unc_edu__Level_3__RSEM_genes_normalized__data.Level_3.2014051800.0.0.tar

5. mRNA:

gdac.broadinstitute.org_OV.Merge_transcriptome__agilentg4502a_07_3__unc_edu__Level_3__unc_lowess_normalization_gene_level__data.Level_3.2014090200.0.0.tar

6. Clinical data:

gdac.broadinstitute.org_OV.Merge_Clinical.Level_1.2014051800.0.0.tar
